# Natural carbon-based dots from humic substances

**DOI:** 10.1038/srep10037

**Published:** 2015-05-06

**Authors:** Yongqiang Dong, Lisi Wan, Jianhua Cai, Qingqing Fang, Yuwu Chi, Guonan Chen

**Affiliations:** 1 College of Chemistry, Ministry of Education Key Laboratory of Analysis and Detection for Food Safety, and Fujian Provincial Key Laboratory of Analysis and Detection for Food Safety, Fuzhou University, Fujian 350108, China

## Abstract

For the first time, abundant natural carbon-based dots were found and studied in humic substances (HS). Four soluble HS including three humic acids (HA) from different sources and one fulvic acids (FA) were synthetically studied. Investigation results indicate that all the four HS contain large quantities of Carbon-based dots. Carbon-based dots are mainly small-sized graphene oxide nano-sheets or oxygen-containing functional group-modified graphene nano-sheets with heights less than 1 nm and lateral sizes less than 100 nm. Carbon-based nanomaterials not only contain abundant *sp*^*2*^-clusters but also a large quantity of surface states, exhibiting unique optical and electric properties, such as excitation-dependent fluorescence, surface states-originated electrochemiluminescence, and strong electron paramagnetic resonance. Optical and electric properties of these natural carbon-based dots have no obvious relationship to their morphologies, but affected greatly by their surface states. Carbon-based dots in the three HS have relative high densities of surface states whereas the FA has the lowest density of surface states, resulting in their different fluorescence properties. The finding of carbon-based dots in HS provides us new insight into HS, and the unique optical properties of these natural carbon-based dots may give HS potential applications in areas such as bio-imaging, bio-medicine, sensing and optoelectronics.

Humic substances (HS) as the most widely-spread natural organic matters are quite important for agriculture^1^,^2^, industry^3^,^4^, environment^5^,^6^, and biomedicine^7^. Although broad attention has been paid to the study of HS, the characterization of complex mixtures of HS is still one of the most important items in HS research nowadays. Great efforts have been made to elucidate the molecular structures of HS. In general, HS are considered to be high molecular weight organic compounds, and several molecular structures have been proposed to describe their structures^8^,^9^,^10^. However, the components and properties of the highly complex substances are still not fully understood. For an example, one would ask whether the complex HS are all composed of those organic compounds. It would be worthy discussing the formation mechanisms and processes of HS. Up to now, some mechanisms have been proposed to explain the formation of HS, such as “sugar-amine condensation” theory, “polyphenol” theory, and the most popular “lignin” theory^11^,^12^. Nevertheless, HS are formed by physical, chemical and biochemical reactions during the decay and transformation of plant and microbial remains, i.e. a process called as humification. Apparently, the humification process is similar in a sense to the coalification process^13^. Furthermore, HS can be obtained from not only soil, water, lake sediments and shales, but also many kinds of coals such as peats, brown coals and lignites. Therefore, the chemical composition of HS should be similar in some degree to that of coals. Recent research results have indicated that coals contain abundant carbon-based nanomaterials, in particular some carbon-based dots^14^,^15^. Therefore, it is reasonable to believe that HS may also contain those carbon-based nanomaterials.

Carbon-based nanomaterials have been hot research topics for many years^16^,^17^,^18^. Carbon-based dots are emerging luminescent carbon-based nanomaterials^19^,^20^. They exhibit unique optical properties such as fluorescence (FL), chemiluminescence (CL) and electrochemiluminescence (ECL) due to their quantum confinement and edge effects^21^,^22^,^23^,^24^,^25^,^26^,^27^,^28^,^29^,^30^. Furthermore, they present many advantages including low toxicity, low cost, robust chemical and optical inertness over those semiconductor based quantum dots^19^,^20^. Accordingly, carbon-based dots have been proposed to have great potential applications in many fields, such as bio-imaging^21^, cell-imaging^22^,^23^, sensing^24^,^25^,^26^,^27^, and photovoltaic devices^28^. Carbon-based dots have been synthesized from various carbon sources using either “bottom-up” or “to-down” methods. However, most of these methods suffer from high cost, low efficiency or complex operation, and are not suit for the production of bulk quantities of carbon-based dots. Apparently, it would be great if carbon-based dots could be obtained from the wide-spread HS. It would be even better if HS could be used directly as carbon-based dots.

In this work, we tried to study systematically the microstructure of HS. Regardless the origin, HS can be divided into three main fractions according to their solubility at different pH values, namely fulvic acids (FA), soluble at all pH values; humic acids (HA), insoluble in water under strong acidic conditions (pH < 2) but soluble in water with higher pH values, especially well soluble in alkaline solutions; and humin, insoluble at all pH values. Obviously, it would be difficult to obtain any distinguishable CNMs directly from humin due to its poor solubility. Herein, four HS including a FA and three HA from different sources were chosen for study. Experimental results indicate that all the investigated HS contain abundant carbon-based nanomaterials, which are primarily various kinds of carbon-based dots. Furthermore, these natural carbon-based dots show unique FL and excellent ECL activities, which are comparable with those of many artificial ones. To the best of our knowledge, this is the first report about the natural carbon-based dots. It would be of significance for the preparation of carbon-based dots. What is more important, it would provide a new insight into the nature of wide-spread HS.

## Results and Discussion

**Figure 1:**
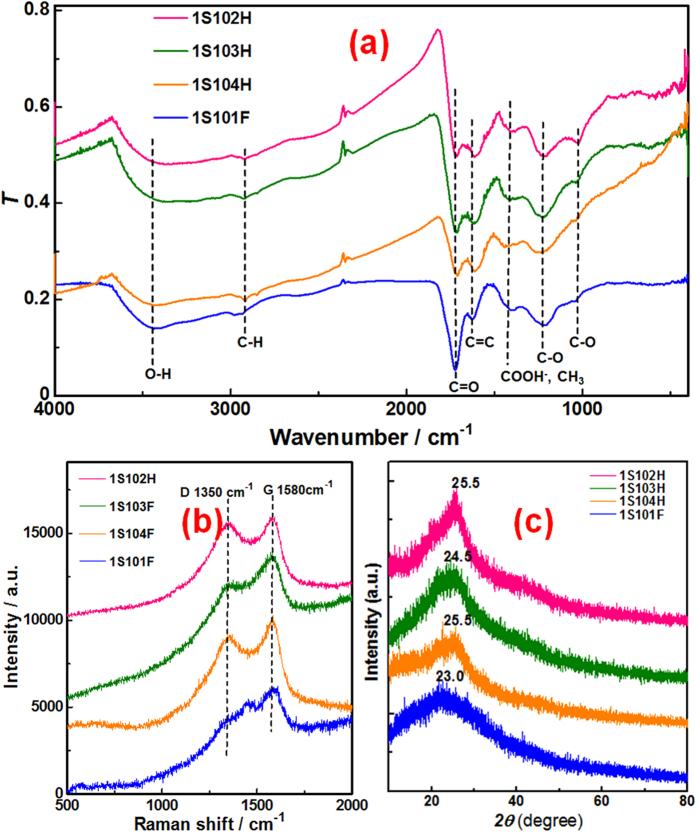
FTIR (**a**) Raman (**b**) and XRD (**c**) spectra of the four HS.

All the four HS (1S102H, 1S103H, 1S104H, 1S101F) are mainly composed of carbon, oxygen and hydrogen atoms, and contain small quantities of nitrogen and sulfur atoms (Supporting information, Table S1). It can be found that 1S101F has the highest oxygen content, implying that 1S101F should contain the most abundant oxygen-containing functional groups. All Fourier transform infrared (FTIR) spectra of the four HS (Fig. 1a) show strong absorption bands of O-H at 3400-3300 cm^−1^, weak absorption bands of C-H at around 2930 and 2840 cm^−1^, sharp absorption bands of C=O and C=C at around 1620 and 1720 cm^−1^, broad absorption bands of COO^−^ and CH_3_ at around 1420 cm^−1^, and broad absorption bands of C-O at around 1240 and 1020 cm^−1^. The FTIR results indicate that all the four HS contain abundant oxygen-containing functional groups (carboxyl, carbonyl and hydroxyl group) and sp^2^ aromatic structure units. Furthermore, the relative absorption intensity of C=O group to C=C group is particularly strong for 1S101F, suggesting that 1S101F should have more abundant carboxyl group than the other three HS. Apparently, the excellent solubility of 1S101F should be related to their high content of carboxyl group. The Raman spectra of the four HS (Fig. 1b) show the characteristic D band at 1350 cm^−1^ and G band at 1580 cm^−1^, which are attributed to the first-order scattering of the *E*_2g_ vibration mode in the graphite sheet and structure defects, respectively^18^. Accordingly, the Raman spectra further confirm that all the four HS should contain abundant graphite-relative structure units. Furthermore, the intensity ratios (*I*_D_/*I*_G_) of the four HS decrease in the order of 1S102H (~0.95), 1S103H (~0.86), 1S104H (~0.84), and 1S101F (~0.57). This means that 1S102H has the highest defect density whereas 1S101F has the lowest defect density. It seems no significant difference between 1S103H and 1S104H from the Raman spectra (more evidences from the Electron Paramagnetic Resonance (EPR) spectra and further discussion about the defect density will be given in the following sections). The X-ray powder diffraction (XRD) studies indicate that all the four HS show a wide peak for carbon-based nanomaterials (Fig. 1c). The peaks of 1S102H and 1S104H center at around 25.5°, corresponding a distance of 3.49 Å, a little larger than the interlayer distance of graphite (3.37 Å). The fact may be related to the abundant oxygen-containing functional groups present in the two HS. For 1S103H and 1S101F, the oxygen content is even further increased (Supporting information, Table S1). Accordingly, the interlayer distances are further increased, 3.63 (2θ = 24.5°) and 3.87 Å (2θ = 23.0°) for 1S103H and 1S101F, respectively.

**Figure 2:**
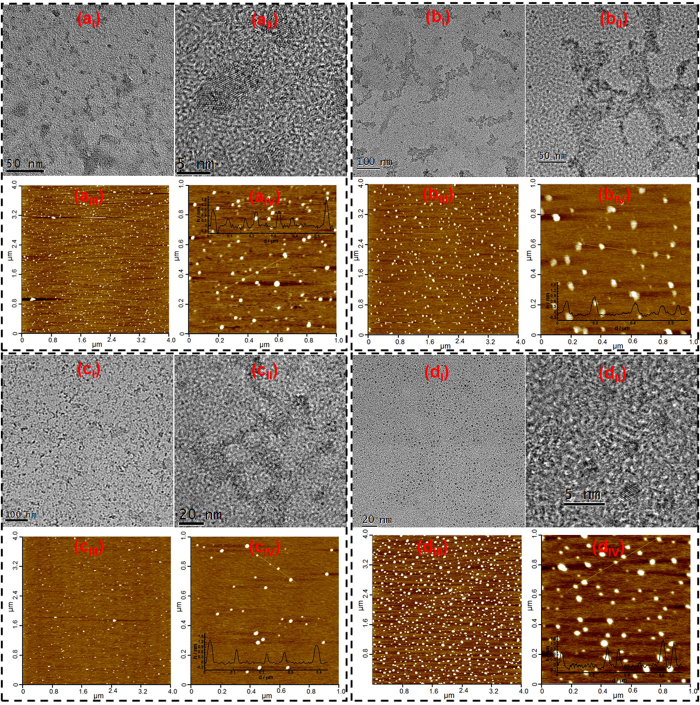
TEM (I), HRTEM (II) and AFM (III, IV) images of 1S102H (**a**), 1S103H (**b**), 1S104H (**c**) and 1S101F (**d**) The insets in a_IV_, b_IV_, c_IV_ and d_IV_ are the height profiles along the lines in the images.

All the characterization results mentioned above are quite similar to those of many previously reported carbon-based nanomaterials (such as carbon-based dots, and few-layer graphene nano-ribbons)^20^,^21^,^22^,^23^. Therefore, it is supposed that all the four HS should contain similar carbon-based nanomaterials. Subsequently, all the four HS were further investigated by transmission electron microscopy (TEM), high resolution TEM (HRTEM) and atomic force microscopy (AFM). TEM image of 1S102H (Fig. 2 aI) shows that the HS contains a large quantity of nano-sheets. Although the nano-sheets are not uniform in size, most of them are less than 10 nm in lateral size. The corresponding HRTEM image (Fig. 2 aII) indicates that most of the nano-sheets have lattice spacings of 0.220 ~ 0.240 nm, agreeing well with that of the (1120) lattice fringes of graphene. The AFM images (Fig. 2 aIII and aIV) demonstrate the topographic morphology of the nano-sheets, the heights of which are range from 0.4 to 2.0 nm with an average value of about 0.8 nm, which is much smaller than the lateral size. Therefore, the carbon-based nanomaterials contained in 1S102H should be mainly oxygen-containing group-modified graphene nano-sheets. The TEM image of 1S103H (Fig. 2 bI) also show many nano-sheets. However, the lateral sizes of these nano-sheets are not so uniform, ranging from several to dozens of nanometers. The HRTEM image (Fig. 2 bII) reveals that these nano-sheets have no obvious lattice structure. The non-crystalline structure may be related to their high oxygen content. AFM characterization indicates that the topographic heights of the nano-sheets are mainly distributed in the range of 0.4 to 1.1 nm with an average value of about 0.6 nm (Fig. 2 bIII and b_IV_), implying that the nano-sheets should be mainly single layer. The results suggest that the carbon-based nanomaterials in 1S103H should be mainly graphene oxide (GO) nano-sheets. TEM and HRTEM images of 1S104H (Fig. 2 cI and c_II_) also show abundant nano-sheets without obvious crystalline structure like 1S103H. The lateral sizes of the nano-sheets are primarily distributed from 10 to 20 nm, which are relative small and uniform when compared with that of the nano-sheets in 1S103H. The topographic heights of the nano-sheets are distributed in the range of 0.4 to 1.3 nm with an average value of about 0.7 nm (Fig. 2 cIII and cIV). Apparently, the carbon-based nanomaterials in 1S104H should also be mainly GO nano-sheets. TEM images show 1S101F contain a large quantity of small dots with uniform lateral size about 2-4 nm (Fig. 2 dI). HRTEM images reveal their lattice spacing of about 0.220 nm (Fig. 2 dII). The AFM images reveal that the heights of those dots range from 0.4 to 1.8 nm, with an average value of about 0.9 nm (Fig. 2 dIII and dIV). Apparently, the carbon-based nanomaterials contained in 1S101F are also some oxygen-containing groups modified graphene nano-sheets, whose lateral sizes are much smaller than those of 1S102H. Actually, besides the abundant small-sized carbon-based nanomaterials, a small quantity of larger-sized carbon-based nanomaterials can be also found in these HS. For example, some relative large-sized nano-sheets with typical crystal characteristic of graphite can also be found from 1S103H and 1S101F (see Fig. S1, S2), and some large-sized GO sheets can also be found from 1S104H (see Fig. S3). However, the relative content of these larger-sized carbon-based nanomaterials are quite low when compared with those small-sized carbon-based nanomaterials from the TEM investigation.

As have been discussed above that all the four studied HS contain abundant small-sized carbon-based nanomaterials, either GO nano-sheets or oxygen-containing group-modified graphene nano-sheets. However, it is necessary to further confirm that these small-sized carbon-based nanomaterials are carbon-based dots, which present unique optical properties due to their quantum confinement and edge effects^31^. Accordingly, the optical properties of the four HS are subsequently investigated.

**Figure 3:**
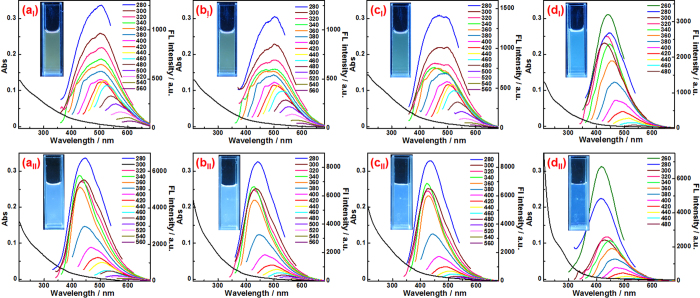
UV-vis absorption (black curves) and FL emission spectra (recorded for progressively increasing excitation wavelengths in 20 nm increments) of 1S102H (**a**), 1S103H (**b**), 1S104H (**c**) and 1S101F (**d**) before (I) and after (II) the reduction by NaBH_4_ The concentration of all the four HS is 0.02 mg/mL. Insets are the photographs of the four HS solutions before and after the reduction of NaBH_4_ under a UV light of 365 nm.

As shown in Fig. 3, all the four HS show a weak UV-vis absorption peak at around 280 nm (black cures in Fig. 3aI to 3dI), representing a typical absorption of an aromatic π system (π-π* transition). The broad absorption with a gradual change up to the long wavelength (~600 nm for the three HA and ~500 nm for the FA) indicates the existence of band tail by defect states^32^. Apparently, the defect density of FA should be much less than those of the three HA (It will be further discussed in the following results). Nevertheless, the UV-vis absorption characteristics of all the four HS fit well those of other reported carbon-based dots, implying that the small-sized carbon-based nanomaterials should be carbon-based dots.

As also shown in Fig. 3, all the four HS have good fluorescence (FL) activities (other curves except for the black one in Fig. 3aI to 3dI), and give green or blue emission under excitation of 365 nm UV light (see the insets of Fig. 3aI to 3dI). Like many reported carbon-based dots^15^,^22^,^24^, the FL spectra of all the HS are excitation-dependent. The maximum emission wavelengths of all the three HA first keep stable at around 505 nm, when the excitation wavelength is increased gradually from 280 nm to 440 nm, then exhibits an obvious red-shift when the excitation wavelength is further increased from 440 to 580 nm. The maximum emission wavelength of FA always red-shifts from 430 to 560 nm when the excitation wavelength is increased gradually from 280 to 500 nm. Moreover, the FL properties of all the four HS are affected by the chemical reduction. When the HS are reduced with NaBH_4_, the UV-vis absorption intensities of all the four HS decrease in the long wavelength region and increase in the short wavelength region (black curves in Fig. 3aII to 3dII), implying the change of the surface states. Correspondingly, the FL behaviors change greatly (other curves except for the black one in Fig. 3aII to 3dII). The main emission spectra of the HS show obvious blue-shift, accompanied by enhancement in FL quantum yields (FLQY) (see Table S2). All these FL properties are quite similar to those of previously reported carbon-based dots^33^,^34^,^35^, further confirming that the small-sized carbon-based nanomaterials are substantially carbon-based dots. Furthermore, the FLQY of these natural carbon-based dots, especially those in 1S101F, is comparable with that of many artificial unpassivated carbon-based dots^22^,^30^,^32^,^34^,^36^. It would be worth discussing the relationship of the FL properties among the four HS. On one hand, the FL behaviors of the three HA are quite similar, from both FL spectra and FLQY. However, the morphologies of the carbon-based dots in the three HA are different, which has been discussed above. That is to say, the morphology of the carbon-based dots in the HA should have no obvious effect on their FL properties. On the other hand, the FL behaviors of FA are quite different from HA. The maximum emission wavelength of the main FL band is about 440 nm, which is much shorter than those of the three HA. Furthermore, the FLQY of FA is obviously higher than those of the three HA. Although the exact FL mechanism of carbon-based dots is still arguable, increasing evidences indicate that the FL of carbon-based dots is related to their surface states^33^,^34^,^35^. Accordingly, the bluer and brighter FL of FA might be related to the fact that the carbon-based dots in FA have the lowest surface state density, which would be further confirmed by the following results.

**Figure 4:**
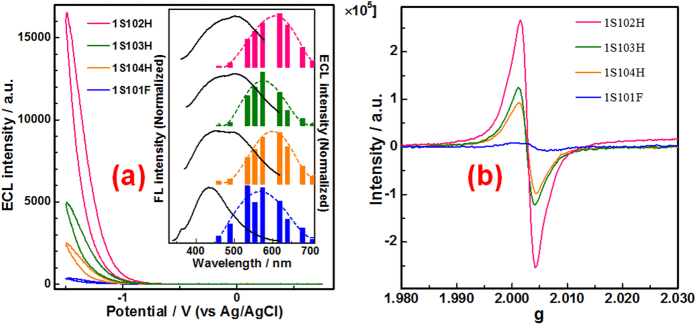
ECL response of the four HS in the presence of 1 mM K_2_S_2_O_8_ (**a**); EPR spectra of the four HS (**b**) Inset in (**a**) shows the main PL band (excited at 320 nm, black lines) and ECL spectra of four HS. Concentration of HS for ECL: 0.5 mg/mL; potential window: −1.5 ~ +0.75 V; scan rate: 0.2 V/s; starting potential: 0 V; initial scan direction: positive; mass of HS for EPR: 40 mg.

Electrochemiluminescence (ECL) is another unique property of carbon-based dots^36^. What is more important, ECL has been proposed to be a powerful technic to study the surface states of various quantum dots^36^,^37^,^38^. Therefore, ECL behaviors of the four HS are investigated subsequently. It should be noted that the addition of a high concentration electrolyte (e.g. 1 M KNO_3_) to decrease the significant uncompensated *i*R drop is necessary for measuring the coreactant-ECL of carbon nanomaterials, which has been discussed in details in our previous work^39^. As shown in Figure S4, all the four HS can produce obvious transient ECL signal when 1 Hz potential steps between +1.8 and −1.5 V are applied. The transient ECL signal could be attributed to the electron transfer reaction between reduced carbon-based dots and oxidized carbon-based dots, which has been well discussed elsewhere^15^,^39^. When the potential is cycled positively from 0 V in the range from +0.75 to −1.5 V, none of the four HS produces any detectable electrochemical response (Fig. S5). Similarly, no observable ECL signal was detected for all the four HS. However, in the presence of 1 mM S_2_O_8_^2−^ as a coreactant, all the four HS produce strong cathodic ECL signal (Fig. 4a). The strong coreactant ECL signal should be caused by the electron transfer annihilation between the reduced carbon-based dots and the electro-generated SO_4_^•–^15,39. The ECL spectra have been also detected. Generally, the ECL spectra of all the four HS show obvious red-shift comparing with their main PL band (The inset in Fig. 4a). The red-shift ECL emission are in good agreement with those of many reported carbon-based dots^15^,^26^,^34^,^36^. These ECL behaviors indicate that those small-sized carbon-based nanomaterials are essentially carbon-based dots. It has been well discussed elsewhere^34^,^36^,^39^, that the ECL properties of carbon based dots should be mainly dependent on the surface states, which may be affected by the functional groups. FTIR spectra indicate that the functional groups in the four HS are quite similar, mainly carboxyl and hydroxyl groups (Fig. 1a). However, the contents of the functional groups in the four samples are different, resulting in a little difference among the four ECL spectra.

Besides a little difference in ECL spectra, the four HS show quite different ECL intensities at a same mass concentration (Fig. 4a). The ECL intensity of carbon based dots should be mainly dependent on density of the surface states. To confirm this viewpoint, all the four HS are further characterized by the EPR spectra, which has been proved to be a powerful technique to investigate the C-related dangling bond centers (so-called defect states or surface states) of carbon-based nanomaterials such as carbon nanotubes^40^, fullerenes and graphene^41^,^42^. Fig. 4b shows the EPR signal of the four HS. As shown, in each case only one EPR signal, of symmetric shape, is observed with corresponding zero-crossing g value of 2.003, which agrees well with that of graphene nanoribbons^43^. These results indicate that all the four HS contain abundant C-related dangling bonds of spin S = 1/2. Furthermore, the EPR intensities of the four HS decrease in the order of 1S102H, 1S103H, 1S104H and 1S101F, reflecting the different spin densities of the four HS. Apparently, the EPR intensities and ECL intensities of the four HS have the same change tendency, implying that the ECL activities of HS should be mainly dependent on their C-related dangling bond centers. In other words, the ECL intensities of the four HS are dependent greatly on the surface state density. To further evaluate the ECL activities of these natural carbon-based dots, ECL efficiencies of the four HS should be measured. Unfortunately, like most reported carbon-based dots^36^,^39^, all the four HS produces no obvious electrochemical signal during the potential scanning (see Figure S5), leading to great difficulty in the calculation of ECL efficiency. Accordingly, the ECL behaviors of the four HS were compared with a kind of high ECL active artificial carbon-based dots obtained from activated carbon under the same condition (see Figure S6)^39^. Apparently, the ECL behavior of the artificial and natural carbon-based dots have no obvious difference, and the ECL intensities of the natural carbon-based dots, especially those in 1S102H, are comparable with that of the artificial carbon-based dots.

In conclusion, all the four studied HS have been found to contain mainly small-sized carbon-based nanomaterials, either GO nano-sheets or graphene nano-sheets modified with oxygen-containing groups. These carbon-based nanomaterials are essentially carbon-based dots, exhibiting unique optical properties, including UV-vis, FL and ECL. The optical properties of these natural carbon-based dots are nearly independent on their morphologies, but affected primarily by their present surface states. This discovery would be significant to not only the study of carbon-based dots, but also the comprehensive understand of HS.

## Methods

### Materials

Four HS including a FA obtained from Suwannee river (1S101F) and three HA obtained respectively from Elliott soil (1S102H), Pahokee peat (1S103H) and Leonardite (1S104H) were purchased from International Humic Substances Society. In order to keep the natural properties of the HS as much as possible, the HS samples were used directly without any treatment. HS solutions for FL measurement were prepared by dissolving HS solid into 0.1 M (pH 7) phosphate buffer solution (PBS). K_2_S_2_O_8_ and NaBH_4_ were purchased from Sigma-Aldrich. All other reagents were of analytical reagent grade and used without further purification. A 0.1 M PBS (pH 7) containing 1 M KNO_3_ was used for the coreactant ECL measurement. Doubly distilled water was used throughout the work.

### Reduction of HS

All the four HS were reduced by NaBH_4_ according to a reported method^35^. In a typical experiment, 5 mL 2 mg mL^−1^ HS solution was heated to 80 °C, followed by the addition of 10 mg NaBH4. The solution was refluxed for 12 h and then cooled to room temperature.

### Sample characterizations

Elemental analysis was carried out using a Vario MICRO organic elemental analyzer. FTIR spectra were recorded on a Thermo Nicolet 360 spectrophotometer. XRD patterns were obtained from a Rigaku D/max-3C (Japan) using Cu Ka radiation. Raman spectra were measured using a Renishaw 1000 microspectrometer (excitation wavelength of 632.8 nm). TEM and HRTEM measurements were performed on a Tecnai G2 F20S-TWIN electronic microscopy at operation voltage of 200 KV. The height distribution of the obtained GQDs was characterized by atomic force microscopy (Nanoman, Veeco, Santa Barbara, CA) by using tapping mode. UV-Vis absorption spectra were recorded by a Lambda 750 UV/Vis spectrophotometer. All FL spectra were obtained by a Cary Eclipse Varian fluorescence spectrophotometer. ECL signals were measured simultaneously by an ECL & EC multi-functional detection system (MPI-E, Remex Electronic Instrument Lt. Co., Xi’an, China) equipped with three electrodes system (a 0.3 cm^2^ Pt wire working electrode, a Pt wire counter electrode and an Ag/AgCl reference electrode). EPR spectra were recorded on a Bruker A-300-EPR X-band spectrometer.

## Author Contributions

Y.D. designed the experiments and wrote the manuscript, L.W. contributed to the experimental design and obtained the AFM, TEM, FTIR and Raman characterizations. J.C. performed the XRD analysis. Q. F. carried out the FL and ECL measurements. Y.C. and G.C. designed, oversaw all phases of the research and corrected the manuscript.

## Additional Information

**How to cite this article**: Dong, Y. *et al.* Natural carbon-based dots from humic substances. *Sci. Rep*. **5**, 10037; doi: 10.1038/srep10037 (2015).

## Supplementary Material

Supplementary Information

## References

[b1] Timothy K. H. & Thomas G. B. Humic substances generally ineffective in improving vegetable crop nutrient uptake or productivity. HortScience 45, 906–910 (2010).

[b2] Islam K. M. S., Schuhmacher A. & Gropp J. M. Humic acid substances in animal agriculture. Pak. J. Nutr. 4, 126–134 (2005).

[b3] Koivula N. & Hänninen K. Biodeterioration of cardboard-based liquid containers collected for fibre reuse. Chemosphere 38, 1873–1887 (1999). 10101851 10.1016/s0045-6535(98)00402-0

[b4] Schmeide K. *et al.* Uranium(VI) sorption onto phyllite and selected minerals in the presence of humic acid. Radiochim. Acta 88, 723–728 (2000).

[b5] Pacheco M. L. & Havel J. Capillary zone electrophoretic (CZE) study of uranium(VI) complexation with humic acids. J. Radioanal. Nucl. Chem. 248, 565–570 (2001).

[b6] Verstraete W. & Devliegher W. Formation of non-bioavailable organic residues in soil: Perspectives for site remediation. Biodegradation 7, 471–485 (1996).

[b7] Klöcking R., Helbig B., Schötz G., Schacke M. & Wutzler P. Anti-HSV-1 activity of synthetic humic acid-like polymers derived from p-diphenolic starting compounds. Antivir. Chem. Chemother. 13, 241–249 (2002). 12495212 10.1177/095632020201300405

[b8] Stenson A. C., Landing W. M., Marshall A. G. & Copper W. T. Ionization and fragmentation of humic substances in electrospray ionization Fourier transform-ion cyclotron resonance mass spectrometry. Anal. Chem. 74, 4397–4409 (2002). 12236348 10.1021/ac020019f

[b9] Stenson A. C., Marshall A. G. & Copper W. T. Exact masses and chemical formulas of individual suwannee river fulvic acids from ultrahigh resolution ESI FT-ICR mass spectra. Anal. Chem. 75, 1275–1284 (2003). 12659186 10.1021/ac026106p

[b10] Kujawinski E. B. *et al.* The application of electrospray ionization mass spectrometry (ESI MS) to the structural characterization of natural organic matter. Org. Geochem. 33, 171–180 (2002).

[b11] In Humic substances: structures, properties and uses (eds Davies G. *et al.* ) Royal Society of chemistry 1998).

[b12] Oglesby R. T., Christman R. F. & Driver C. H. The biotransformation of lignin to humus facts and postulates. Adv. Appl. Microbiol. 9, 171–184 (1967). 4866748

[b13] Given P. H. In Coal Science (eds Gorbaty M. L. *et al.* ) Vol. 3, 63–252 Orlando Academic press 1984).

[b14] Ye R. *et al.* Coal as an abundant source of graphene quantum dots. Nat. Commun. 4, 2943 (2013). 24309588 10.1038/ncomms3943

[b15] Dong Y. *et al.* Graphene quantum dots, graphene oxide, carbon quantum dots and graphite nanocrystals in coals. Nanoscale 6, 7410–7415 (2014). 24875280 10.1039/c4nr01482k

[b16] Kroto H. W., Heath J. R., O’Brien S. C., Curl R. F. & Smalley R. E. C60: Buckminsterfullerene. Nature 318, 162–163 (1985).

[b17] Iijima S. Helical microtubules of graphitic carbon. Nature 354, 56–58 (1991).

[b18] Zhu Y. *et al.* Graphene and graphene oxide: synthesis, properties, and applications. Adv. Mater. 22, 3906–3924 (2010). 20706983 10.1002/adma.201001068

[b19] Baker S. N. & Baker G. A. Luminescent Carbon Nanodots: Emergent Nanolights. Angew. Chem., Int. Ed. 49, 6726–6744 (2010). 10.1002/anie.200906623 20687055

[b20] Zhang Z., Zhang J., Chen N. & Qu L. Graphene quantum dots: an emerging material for energy-related applications and beyond. Energy Environ. Sci. 5, 8869–8890 (2012).

[b21] Zhu S. *et al.* Surface chemistry routes to modulate the photoluminescence of graphene quantum dots: from fluorescence mechanism to up-conversion bioimaging applications. Adv. Funct. Mater. 22, 4732–4740 (2012).

[b22] Dong Y. *et al.* One-step and high yield simultaneous preparation of single- and multi-layer graphene quantum dots from CX-72 carbon black. J. Mater. Chem. 22, 8764–8766 (2012).

[b23] Yang Y. T. *et al.* Carbon dots for optical imaging in vivo. J. Am. Chem. Soc. 131, 11308–11309 (2009). 19722643 10.1021/ja904843x PMC2739123

[b24] Dong Y. *et al.* Graphene Quantum Dot as a Green and Facile Sensor for Free Chlorine in Drinking Water. Anal. Chem. 84, 8378–8382 (2012). 22957474 10.1021/ac301945z

[b25] Qu Q., Zhu A., Shao X., Shi G. & Tian Y. Development of a carbon quantum dots-based fluorescent Cu^2+^ probe suitable for living cell imaging. Chem. Commun. 48, 5473–5475 (2012). 10.1039/c2cc31000g 22540124

[b26] Dong Y., Tian W., Ren S., Dai R., Chi Y. & Chen G. Graphene quantum dots/l-cysteine coreactant electrochemiluminescence system and its application in sensing lead(II) Ions. ACS Appl. Mater. Interfaces 6, 1646–1651 (2014). 24405119 10.1021/am404552s

[b27] Bai J., Zhang L., Liang R. & Qiu J. Graphene quantum dots combined with europium ions as photoluminescent probes for phosphate sensing. Chem. Eur. J. 19, 3822–3826 (2013). 23420738 10.1002/chem.201204295

[b28] Yan X., Cui X., Li B. & Li L. Large, solution-processable graphene quantum dots as light absorbers for photovoltaics. Nano Lett. 10, 1869–1873 (2010). 20377198 10.1021/nl101060h

[b29] Dong Y. *et al.* Blue Luminescent Graphene quantum dots and graphene oxide prepared by tuning the carbonization degree of citric acid. Carbon 50, 4738–4743 (2012).

[b30] Liu R., Wu D., Feng X. & Müllen K. Bottom-up fabrication of photoluminescent graphene quantum dots with uniform morphology. J. Am. Chem. Soc. 133, 15221–15223 (2011). 21894989 10.1021/ja204953k

[b31] Ponomarenko L. A. *et al.* Chaotic dirac billiard in graphene quantum dots. Science 320, 356–358 (2008). 18420930 10.1126/science.1154663

[b32] Liu F. *et al.* facile synthetic method for pristine graphene quantum dots and graphene oxide quantum dots: origin of blue and green luminescence. Adv. Mater. 25, 3657–3662 (2013). 23712762 10.1002/adma.201300233

[b33] Zhu S. *et al.* Surface chemistry routes to modulate the photoluminescence of graphene quantum dots: from fluorescence mechanism to up-conversion bioimaging applications. Adv. Funct. Mater. 22, 4732–4740 (2012).

[b34] Li L. *et al.* A Facile microwave avenue to electrochemiluminescent two-color graphene quantum dots. Adv. Funct. Mater. 22, 2971–2979 (2012).

[b35] Zheng H. *et al.* Enhancing the luminescence of carbon dots with a reduction pathway. Chem. Commun. 47, 10650–10652 (2011). 10.1039/c1cc14741b 21879050

[b36] Zheng L., Chi Y., Dong Y., Lin J. & Wang B. Electrochemiluminescence of water-soluble carbon nanocrystals released electrochemically from graphite. J. Am. Chem. Soc. 131, 4564–4565 (2009). 19296587 10.1021/ja809073f

[b37] Ding Z. *et al.* Electrochemistry and electrogenerated chemiluminescence from silicon nanocrystal quantum dots. Science 296, 1293–1297 (2002). 12016309 10.1126/science.1069336

[b38] Bae Y., Myung N. & Bard A. J. Electrochemistry and electrogenerated chemiluminescence of CdTe nanoparticles. Nano Lett. 4, 1153–1161 (2004).

[b39] Dong Y. *et al.* Extraction of electrochemiluminescent oxidized carbon quantum dots from activated carbon. Chem. Mater. 22, 5895–5899 (2010).

[b40] Thess A. *et al.* Crystalline ropes of metallic carbon nanotubes. Science 273, 483–487 (1996). 8662534 10.1126/science.273.5274.483

[b41] Krusic P. J., Roe D. C., Johnston E., Morton J. R. & Preston K. F. EPR study of hindered internal rotation in alkyl-fullerene (C60) radicals. J. Phys. Chem. 97, 1736–1738 (1993).

[b42] Su C. *et al.* Probing the catalytic activity of porous graphene oxide and the origin of this behaviour. Nature Commun. 3, 1298 (2012). 23250428 10.1038/ncomms2315

[b43] Rao S. S., Stesmans A., Kosynkin D. V., Higginbotham A. & Tour J. M. Paramagnetic centers in graphene nanoribbons prepared from longitudinal unzipping of carbon nanotubes. New J. Phys. 13, 113004 (2011).

