# Supplementary material for: Natural carbon-based dots from humic substances
Source: Sci Rep. 2015 May 6;5:10037. doi: 10.1038/srep10037 (PMC4421865; doi:10.1038/srep10037)
Supplement: Supplementary Information [file srep10037-s1.doc]

Supporting Information

Natural carbon based dots from humic substances

Yongqiang Dong, Lisi Wan, Jianhua Cai, Qingqing Fang, Yuwu Chi*, and Guonan Chen

Ministry of Education Key Laboratory of Analysis and Detection for Food Safety, Fujian Provincial Key Laboratory of Analysis and Detection for Food Safety, and Department of Chemistry, Fuzhou University, Fujian 350108, China

Email: y.w.chi@fzu.edu.cn; Tel/Fax: +86-591-22866137.

**Electrochemiluminescnece (ECL) measurement:** ECL experiments were carried out on an ECL detection system (MPI-E, Remex Electroic Instrument Ltd. Co., Xi’an, China) equipped with a home-made ECL cell, which has been described in detail elsewhere [1]. ECL transients of the four HS, without any coreactant, were measured by applying 1 Hz potential steps between +1.8 and -1.5 V. The ECL spectra were obtained by collecting the ECL peak intensity during the cyclic potential sweep with a series of optical filters at 460, 490, 535, 555, 575, 620, 640, 680 and 705 nm.

**Table S1. Elemental analysis of humic substances.**

| Coals | C(%) | H(%) | N(%) | O(%) | S(%) | Ash(%) |
| --- | --- | --- | --- | --- | --- | --- |
| 1S101F | 52.44 | 4.31 | 0.72 | 42.20 | 0.44 | 0.46 |
| 1S102H | 58.13 | 3.68 | 4.14 | 34.08 | 0.44 | 0.88 |
| 1S103H | 56.37 | 3.82 | 3.69 | 37.34 | 0.71 | 1.12 |
| 1S104H | 63.81 | 3.70 | 1.23 | 31.27 | 0.76 | 2.58 |

**Table S2. FLQY of the four HS before and after reduced with NaBH_4_.**

| GQDs | 1S102H | 1S103H | 1S104H | 1S101F |
| --- | --- | --- | --- | --- |
| FLQY (%) before reduction | 0.68 | 0.77 | 0.78 | 1.45 |
| FLQY (%) after reduction | 1.59 | 1.51 | 1.76 | 3.71 |


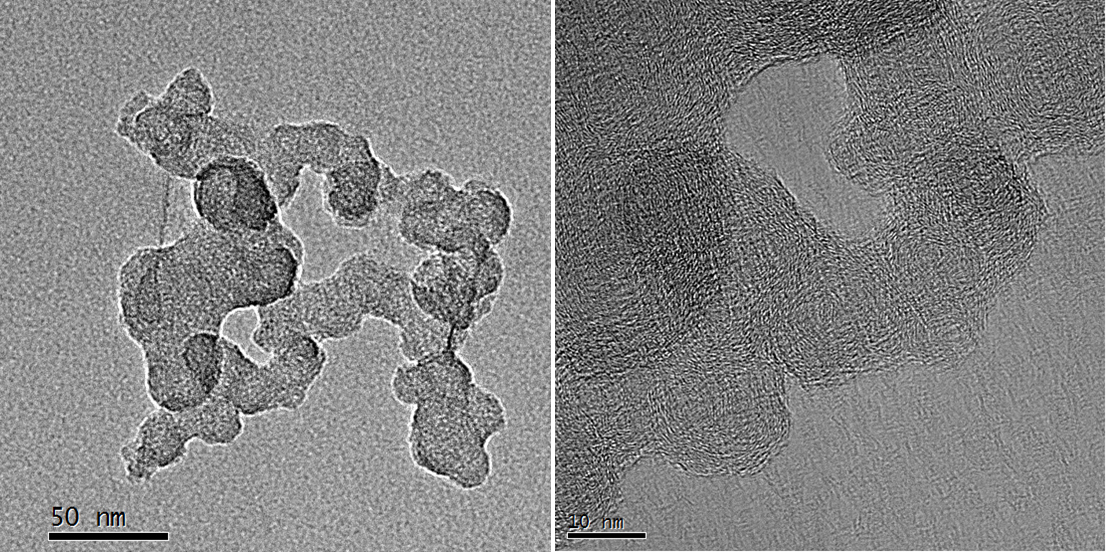


**Figure S1.** TEM and HRTEM images of the larger-sized CNMs in 1S103H.


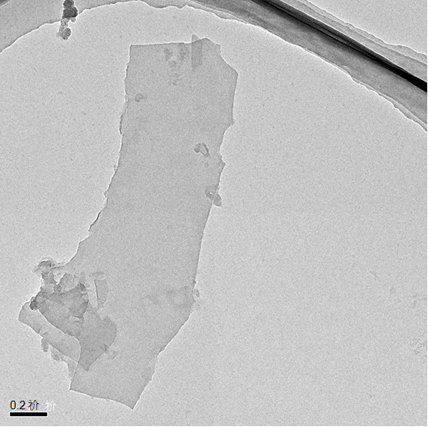


**Figure S2.** TEM image of the larger-sized CNMs in 1S104H.


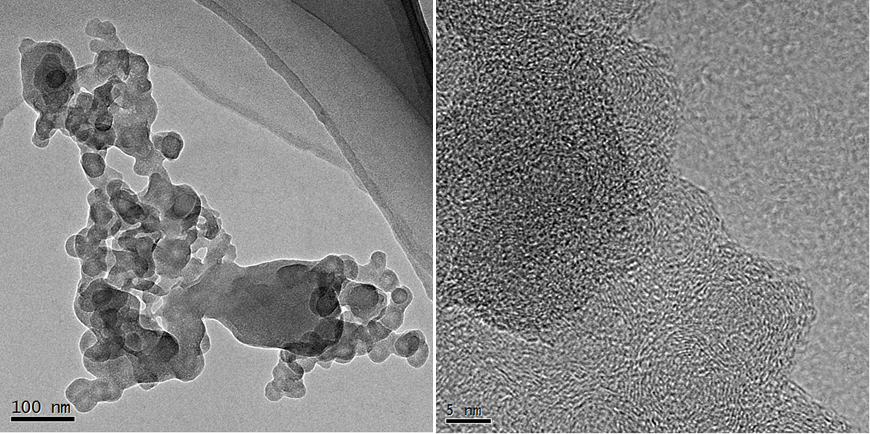


**Figure S3.** TEM and HRTEM images of the larger-sized CNMs in 1S101F.


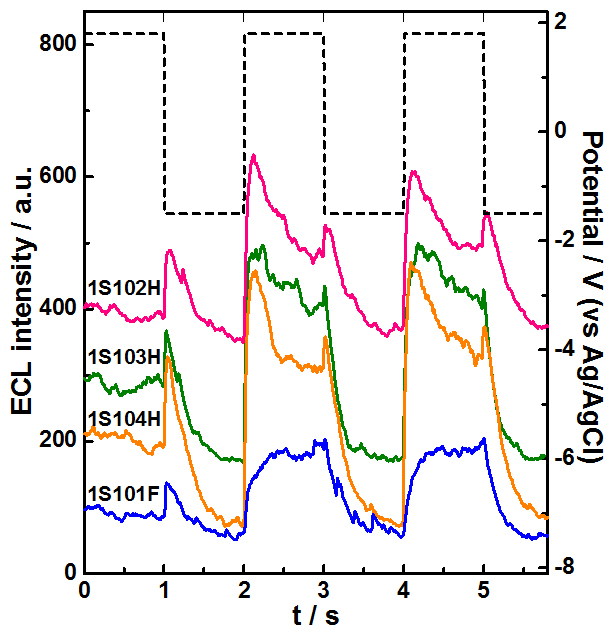


**Figure S4.** ECL transients (lower curves) by stepping potential (upper curve) between -1.5 and +1.8 V of the four HS.


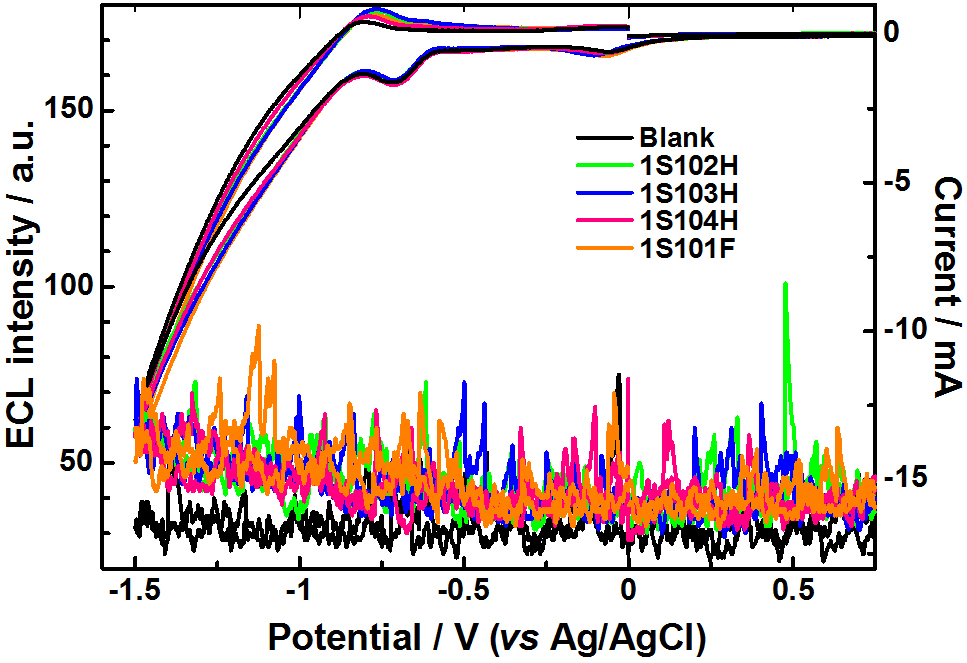


**Figure S5.** ECL (Lower curves) and electrochemical (Upper curves) responses for PBS blank and the four HS in pH 7 PBS (0.1 mol L^-1^ phosphate + 1 mol L^-1^ KNO_3_). Concentration of HS: 0.5 mg/mL; potential window: -1.5 ~ +0.75 V; scan rate: 0.2 V/s; starting potential: 0 V; initial scan direction: positive.

**Figure S6.** ECL (Lower curve) and electrochemical (Upper curve) responses for artificial carbon-based dots obtained from activated crbon in pH 7 PBS (0.1 mol L^-1^ phosphate + 1 mol L^-1^ KNO_3_). Concentration of the carbon-based dots: 0.5 mg/mL; potential window: -1.5 ~ +0.75 V; scan rate: 0.2 V/s; starting potential: 0 V; initial scan direction: positive.

**Reference**

(S1) Dong, Y. et al. Electrochemiluminescence emission from carbon quantum dot-sulfite coreactant system. *Carbon* **56**, 12-17 (2013).
